# Comparative Evaluation of Airway Assessment Parameters for Predicting Difficult Intubation: A Prospective Observational Study

**DOI:** 10.7759/cureus.101087

**Published:** 2026-01-08

**Authors:** Vignesh Raju N, Kannan N, Sathyasuba Meenakshisundaram, Srinidhi Narayanan

**Affiliations:** 1 Anesthesiology, Sree Balaji Medical College and Hospital, Chennai, IND

**Keywords:** aasi, difficult intubation, mallampati, rhtmd, sternomental distance

## Abstract

Background: Difficult intubation remains a significant concern in anesthetic practice because of its association with airway trauma, hypoxemia, and increased perioperative morbidity. Although multiple bedside airway assessment tests are routinely employed, their predictive accuracy varies, and no single parameter has proven universally reliable. This underscores the need for comparative evaluation of commonly used predictors in specific clinical populations. This study aimed to evaluate and compare the diagnostic accuracy of the modified Mallampati classification (MPC), sternomental distance (SMD), and the ratio of height to thyromental distance (RHTMD) in predicting difficult intubation, using the Intubation Difficulty Scale (IDS) as the reference standard.

Methods: A prospective observational study was conducted among 57 adult patients undergoing elective surgical procedures under general anesthesia with tracheal intubation. Preoperative airway assessment included MPC, SMD, and RHTMD measurements. Laryngoscopy and intubation were performed using standard techniques, and intubation difficulty was graded using the IDS. An IDS score > 5 was considered indicative of difficult intubation. Statistical analysis comprised receiver operating characteristic (ROC) curve analysis with area under the ROC curve (AUC), logistic regression to estimate odds ratios (ORs), and correlation testing to assess associations between predictors and difficult intubation.

Results: The incidence of difficult intubation in the study population was 12 (21.1%). Among the three parameters, RHTMD demonstrated the highest predictive accuracy, with an AUC of 0.84, an adjusted odds ratio (aOR) of 6.5, and a statistically significant association (p < 0.001). MPC also showed a significant predictive value (aOR 4.2, p = 0.018), followed by SMD (aOR 3.8, p = 0.032). ROC analysis confirmed the superior discriminatory ability of RHTMD compared to MPC and SMD. RHTMD had the strongest correlation (r = 0.52), followed by MPC (r = 0.41) and SMD (r = 0.38). Patients with RHTMD ≥ 25 were at significantly increased risk of difficult intubation.

Conclusion: RHTMD emerged as the most accurate single predictor of difficult intubation in this cohort. However, combining multiple airway assessment parameters improved diagnostic reliability. A multimodal preoperative airway assessment strategy may enhance patient safety and reduce the risk of unanticipated difficult intubation.

## Introduction

The prediction of a difficult airway has long been recognised as one of the most critical priorities in anesthetic practice, as failure to anticipate complications during intubation can result in significant morbidity and even mortality. A difficult airway encompasses a spectrum of clinical scenarios in which an anesthesiologist encounters difficulty in establishing or maintaining effective ventilation and oxygenation. Airway patency and gas exchange are typically achieved by three methods: face mask ventilation, supraglottic airway (SGA) devices such as the laryngeal mask airway, and tracheal intubation. Mask ventilation relies on maintaining upper airway patency with maneuvers or adjuncts, whereas SGAs bypass the oropharynx without traversing the vocal cords. Tracheal intubation provides definitive airway continuity through the cords into the trachea. Difficult airway includes difficulty or failure in mask ventilation, SGA placement or ventilation, laryngoscopy, and tracheal intubation using direct, video, or flexible techniques. Unanticipated difficult intubation is associated with hypoxemia, airway trauma, aspiration, prolonged operative time, and, in severe cases, brain damage and cardiac arrest, making early identification and preparation essential for patient safety [1]. Over the years, clinicians have relied on various bedside screening tests such as the modified Mallampati classification (MPC) and sternomental distance (SMD), which were initially introduced as simple clinical tools to stratify airway risk. Although these measures are widely applied, their performance has been inconsistent, with studies indicating relatively low sensitivity and specificity, particularly when used as isolated predictors in diverse patient groups [2,3]. This inconsistency underscores the need for more robust and reproducible parameters that can improve diagnostic accuracy and reduce the incidence of unanticipated events. The Intubation Difficulty Scale (IDS) introduced by Adnet and associates is a numeric score indicating overall intubation difficulty based on seven descriptors associated with intubation difficulty: number of supplementary intubation attempts, number of supplementary operators, alternative techniques used, laryngoscopic grade, subjective lifting force, the use of external laryngeal manipulation, and the characteristics of the vocal cords [2,4].

In an attempt to overcome the limitations of conventional indices, newer anthropometric measurements such as the ratio of height to thyromental distance (RHTMD) and the acromio-axillo-suprasternal notch index (AASI) have been developed. These parameters were designed to incorporate both anatomical proportion and functional alignment, thereby offering a more comprehensive reflection of the airway space than simple linear distances [5]. RHTMD, in particular, has gained attention for its improved ability to differentiate between easy and difficult intubations by adjusting the thyromental distance for patient height, thus reducing inter-individual variability [6]. Similarly, AASI has been suggested as a supplementary measure to capture subtle differences in craniofacial structure that may influence laryngoscopic view, although evidence regarding its effectiveness remains mixed. Comparative trials and meta-analyses have suggested that these newer indices may outperform traditional predictors in selected populations, but their utility has yet to be universally confirmed [7,8].

Despite the availability of a growing body of literature, there remain important gaps in the validation of these predictors within the Indian population. Anatomical variations, demographic differences, and the burden of comorbidities specific to regional cohorts mean that findings from Western studies cannot always be extrapolated directly to Indian patients [9]. Moreover, many of the earlier studies were limited by small sample sizes, heterogeneous inclusion criteria, and inconsistent definitions of difficult intubation, which have led to variations in reported diagnostic accuracy. In this context, there is a clear need for focused studies that examine the predictive value of these indices in Indian cohorts, not only to refine clinical practice but also to provide data that are relevant to local training and guideline development [10,11].

The present study was therefore undertaken with the primary aim of evaluating and comparing the accuracy of MPC, SMD, RHTMD, and AASI in predicting difficult intubation using the IDS as a reference standard. By systematically analysing these indices within a defined cohort of elective surgical patients, the study seeks to provide evidence on which parameters are most useful for routine clinical screening, with the ultimate objective of enhancing patient safety through improved preoperative airway assessment.

## Materials and methods

Study type and setting

This prospective observational study was conducted in the Department of Anesthesiology, Sree Balaji Medical College and Hospital, Chennai. The institution is a tertiary care teaching hospital catering to a broad patient population undergoing elective surgical procedures, thereby providing an appropriate setting for evaluating preoperative airway assessment tools. Ethical approval was obtained from the Institutional Human Ethics Committee prior to commencement of the study (002/SBMCH/IHEC/2023/2086), and written informed consent was secured from all participants in accordance with institutional and national research guidelines.

Study population

The study enrolled 57 adult patients scheduled for elective surgeries under general anesthesia requiring endotracheal intubation. The study duration spanned a predefined period, during which patients presenting for elective procedures were screened consecutively for eligibility.

Inclusion criteria comprised adults aged 18-65 years with American Society of Anesthesiologists (ASA) physical status I or II, as defined by the ASA classification system [12]. ASA I patients (normal healthy patients) have no systemic disease, while ASA II patients have mild systemic disease without functional limitation. Exclusion criteria were established to avoid confounding anatomical or physiological factors that could independently influence intubation difficulty. Patients were excluded if they had a body mass index of 30-40 kg/m^2^ (obesity), pregnancy, or presented with maxillofacial injury, cervical spine trauma, head and neck pathologies, emergency surgery status, or ASA grade III or above. Patients with congenital craniofacial abnormalities, restricted mouth opening, or prior head-and-neck surgeries were also excluded.

Sample size calculation

Sample size estimation was performed using parameters relevant to diagnostic accuracy studies. The formula used was based on the area under the receiver operating characteristic (ROC) curve (AUC):

$AUC = \frac{S_0 - \frac{n_0 (n_0 + 1)}{2}}{n_1 n_0}$

where n₀ and n₁ denote the number of negative and positive cases, respectively, and S₀ represents the sum of ranks for positive cases. This study targeted a total sample size N = 57, with a type I error (α) of 0.05, a type II error (β) of 0.20 (power = 80%), an expected AUC of 0.725, and a null hypothesis AUC of 0.5. A ratio of 2:1 for negative-to-positive class distribution was assumed, consistent with reported incidences of difficult intubation in the general population. These values yielded a statistically adequate sample size for detecting clinically meaningful differences between airway assessment tools.

Preoperative airway assessment

The data were collected using a structured questionnaire that included all recorded airway and anthropometric parameters (Appendix A). All patients underwent comprehensive preoperative airway evaluation by trained anesthesiologists to ensure uniformity and minimize interobserver variability. Four airway assessment parameters were examined: MPC, SMD, RHTMD, and AASI. All tools used were freely accessible and required no specialized proprietary software or equipment.

MPC

The MPC was assessed with the patient seated upright, mouth maximally opened, and tongue fully protruded without phonation, as described in the original classification system [13]. Visibility of oropharyngeal structures was graded from Class I to IV: Class I - Faucial pillars, uvula, and soft palate visualized; Class II - Base of the uvula and soft palate visualized; Class III - Soft palate only visualized; Class IV - Hard palate only visualized.

SMD

SMD was measured as the straight-line distance between the sternum (suprasternal notch) and mentum with the patient’s head fully extended and mouth closed [14]. Shorter distances, less than 12.5 cm, are associated with difficult intubation, suggesting limited atlanto-occipital extension and potential difficulty during laryngoscopy.

RHTMD

RHTMD was calculated by dividing the patient's height (in cm) by the measured thyromental distance (TMD) (in cm) [15]. This ratio normalizes airway evaluation across body habitus and has shown predictive value for difficult intubation. A TMD of less than 6.5 cm (three fingerbreadths), as measured from the thyroid notch to the lower border of the mentum, is indicative of reduced mandibular space and may predict difficulty with intubation.

AASI

AASI represents an anthropometric index reflecting upper thoracic anatomy and neck mobility. The measurement was derived using standard anthropometric techniques described by Nasr-Esfahani et al. [16], capturing the spatial relationships between the acromion, axilla, and suprasternal notch. All airway assessments were performed preoperatively during routine evaluation, and values were documented in structured case-record forms. The absence of cost or licensing requirements for these tools enhanced their feasibility for clinical practice.

Intubation procedure and outcome measurement

Standard anesthetic induction protocols were followed for all patients. After preoxygenation, patients were administered induction agents and neuromuscular blockade as per institutional guidelines. Laryngoscopy was performed using a standard Macintosh blade (Rüsch®, Teleflex Medical, Wayne, USA), and endotracheal intubation was attempted by an anesthesiologist with a minimum of two years of experience. Difficult intubation was defined and quantified using the IDS introduced by Adnet et al. [4], which incorporates seven weighted variables, including the number of attempts, number of operators, alternative techniques used, glottic exposure, lifting force, external laryngeal manipulation, and vocal cord position. IDS values were recorded immediately after securing the airway, with IDS score > 5 categorised as difficult intubation.

Statistical analysis

Data were analysed using IBM SPSS Statistics version 26 (IBM Corp., Armonk, USA) and R statistical software (R Foundation for Statistical Computing, Vienna, Austria). Descriptive statistics, including mean ± standard deviation or median with interquartile range for continuous variables, based on normality using Shapiro-Wilk and Q-Q plots, and percentages for categorical variables, summarized baseline demographic and clinical characteristics. The association between airway parameters and difficult intubation was examined using ROC curve analysis. AUC values were calculated for each airway assessment tool, with AUC > 0.7 indicative of good predictive performance.

Multivariate logistic regression models identified independent predictors of difficult intubation. Odds ratios (ORs) and 95% confidence intervals (CIs) were generated, and statistical significance was defined as p < 0.05. Pearson’s/Spearman's correlation tests assessed relationships between continuous airway parameters and IDS scores, enabling evaluation of linear associations between anatomical measurements and intubation difficulty.

## Results

Table 1 presents the demographic characteristics of the 57 participants enrolled in the study. The age distribution showed that 16 (28.07%) participants were between 18-30 years, 17 (29.83%) were between 31-40 years, 15 (26.31%) were between 41-50 years, and nine (15.79%) were between 51-65 years. Gender distribution was nearly equal, with 28 (49.12%) male participants and 29 (50.88%) female participants. Regarding ASA physical status classification, the majority of participants were classified as ASA I (37 participants, 64.91%), while 20 (35.09%) belonged to ASA II.

**Table 1:** Demographic characteristics of the participants (N = 57) ASA: American Society of Anesthesiologists

Characteristic	N	%
Age (years)		
18-30	16	28.07
31-40	17	29.83
41-50	15	26.31
51-65	9	15.79
Total	57	100.00
Gender		
Male	28	49.12
Female	29	50.88
Total	57	100.00
ASA classification [12]		
ASA I	37	64.91
ASA II	20	35.09
Total	57	100.00

Assessment of intubation difficulty using IDS [4] revealed that 45 patients (78.9%) had easy intubation, while 12 patients (21.1%) experienced mild to moderate difficulty. None of the cases met the criteria for impossible intubation (Table 2). Thus, the overall incidence of difficult intubation in this cohort was 21.1%.

**Table 2:** IDS classification and incidence of difficult intubation (N = 57) IDS: Intubation Difficulty Scale

IDS score category [4]	Description	Number of patients, n (%)
Score = 0	Easy intubation	45 (78.9)
Score < 5	Mild to moderate difficulty	12 (21.1)
Score > 5	Impossible intubation	0 (0.0)

**Figure 1:**
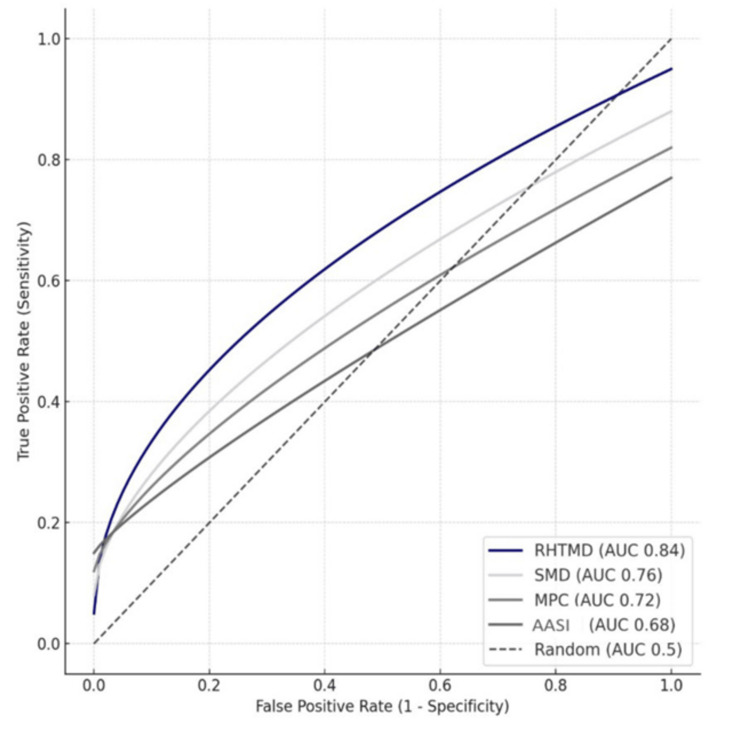
Area under the ROC curve (AUC) for predictive parameters MPC: Mallampati classification; SMD: Sternomental distance; RHTMD: Ratio of height to thyromental distance; AASI: Acromio-axillo-suprasternal notch index Receiver operating characteristic (ROC) curve analysis done. [13-16]

ROC curve analysis was performed to determine the diagnostic accuracy of the four bedside airway predictors. The AUC values indicated that RHTMD [15] was the most accurate single parameter with an AUC of 0.84 (95% CI: 0.75-0.93, p < 0.001). SMD [14] also demonstrated good predictive value (AUC 0.76, 95% CI: 0.65-0.87, p = 0.003), followed by MPC (AUC 0.72, 95% CI: 0.61-0.83, p = 0.008). AASI was the weakest performer, with a lower AUC of 0.68 (95% CI: 0.55-0.81, p = 0.023) (Table 3, Figure 1). These findings confirm that RHTMD [15] offered the highest discrimination for predicting difficult intubation compared to the other indices.

**Table 3:** Area under the ROC curve (AUC) for predictive airway parameters MPC: Mallampati classification; SMD: Sternomental distance; RHTMD: Ratio of height to thyromental distance; AASI: Acromio-axillo-suprasternal notch index Receiver operating characteristic (ROC) curve analysis done. *p-value < 0.05 indicates statistical significance.

Parameter	AUC (95% CI)	p-value
MPC [13]	0.72 (0.61-0.83)	0.008*
SMD [14]	0.76 (0.65-0.87)	0.003*
RHTMD [15]	0.84 (0.75-0.93)	<0.001*
AASI [16]	0.68 (0.55-0.81)	0.023*

**Figure 2:**
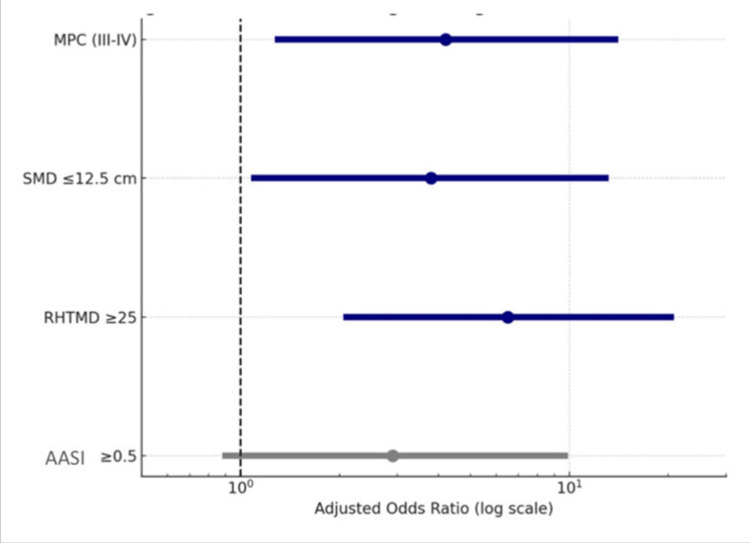
Multivariate logistic regression analysis MPC: Mallampati classification; SMD: Sternomental distance; RHTMD: Ratio of height to thyromental distance; AASI: Acromio-axillo-suprasternal notch index [13-16]

Multivariate logistic regression analysis further supported these findings (Figure 2). An RHTMD ≥ 25 emerged as the strongest independent predictor of difficult intubation, with an adjusted odds ratio (aOR) of 6.5 (95% CI: 2.1-20.1; p < 0.001). Modified MPC grades III-IV were also significantly associated with difficult intubation (aOR 4.2; 95% CI: 1.3-13.6; p = 0.018), as was SMD ≤ 12.5 cm (aOR 3.8; 95% CI: 1.1-12.9; p = 0.032). In contrast, an AASI ≥ 0.5 did not demonstrate a statistically significant association with difficult intubation (aOR 1.6; 95% CI: 0.5-5.1; p = 0.41), suggesting limited predictive utility in this cohort.

**Figure 3:**
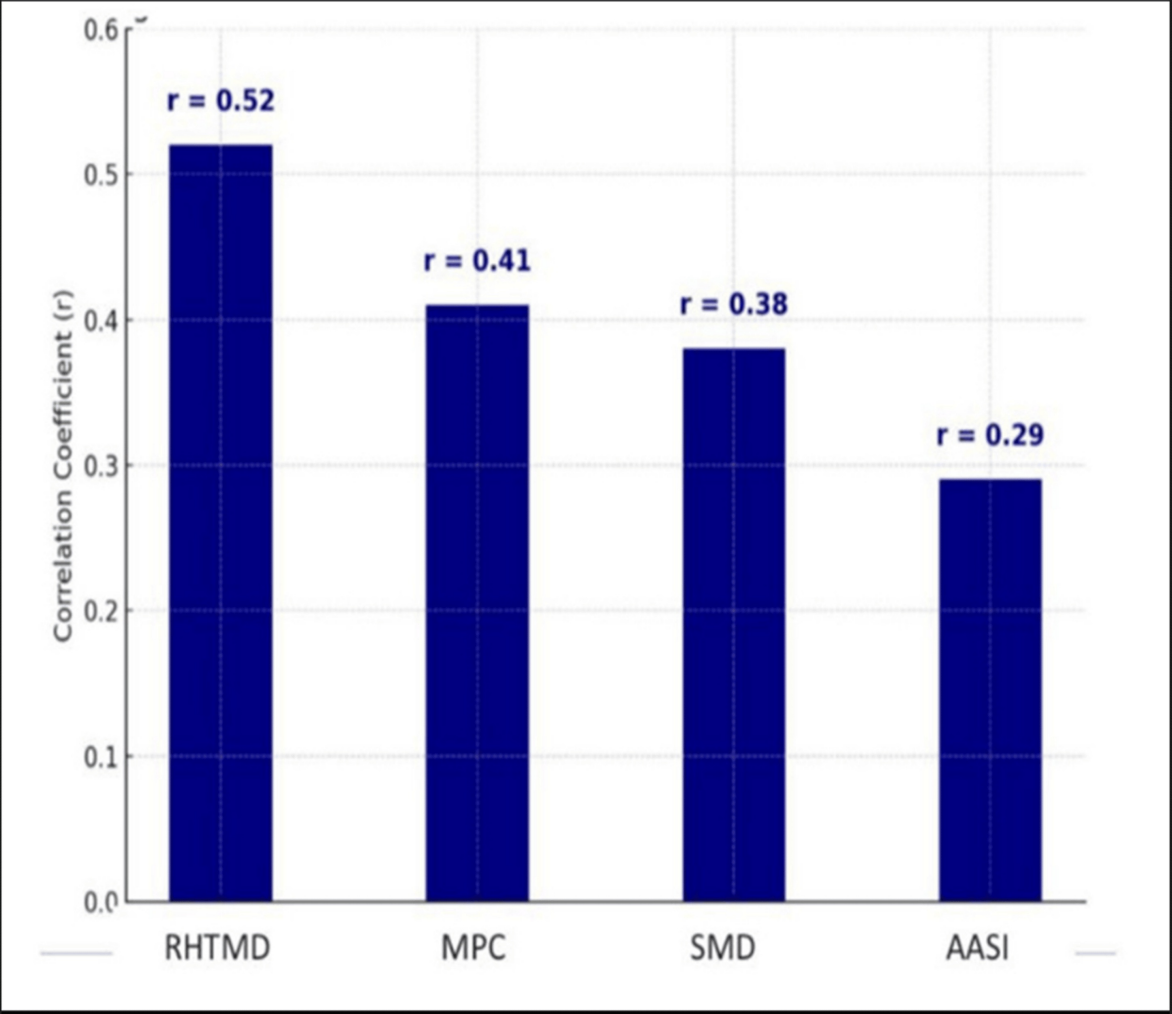
Correlation between parameters and difficult intubation MPC: Mallampati classification; SMD: Sternomental distance; RHTMD: Ratio of height to thyromental distance; AASI: Acromio-axillo-suprasternal notch index Pearson correlation coefficient test done. [13-16]

Shapiro-Wilk testing demonstrated that the continuous airway assessment parameters followed a normal distribution. The modified MPC-derived scores (W = 0.97, p = 0.21), SMD (W = 0.96, p = 0.14), and RHTMD (W = 0.98, p = 0.32) did not significantly deviate from normality. Visual inspection of histograms and Q-Q plots corroborated these findings. Pearson's correlation analysis between the four predictors and IDS scores (Figure 3) demonstrated that RHTMD had the strongest correlation (r = 0.52), followed by MPC (r = 0.41) and SMD (r = 0.38). AASI showed only a weak correlation (r = 0.29), reinforcing the conclusion that it is less reliable for clinical application in this population.

## Discussion

The present study highlights the significance of the RHTMD as the most dependable single predictor of difficult intubation in the examined population. When compared to other bedside assessments such as the modified MPC and SMD, RHTMD consistently demonstrated higher discriminatory accuracy, reflected in superior area under the curve values and stronger ORs. The AASI, though considered in recent years as a novel measure of airway space, showed a weaker association with intubation difficulty in this cohort, suggesting limited reliability when used in isolation [17]. These findings affirm the clinical utility of RHTMD and reinforce the principle that while traditional indices remain useful, newer anthropometric adjustments provide a more robust framework for anticipating difficult laryngoscopy.

The clinical implications of these findings are considerable, particularly given the well-documented risks of unanticipated difficult intubation, including hypoxemia, aspiration, airway trauma, and perioperative morbidity. By prioritizing the use of RHTMD alongside traditional indices, anesthesiologists may enhance their ability to identify at-risk patients and adopt appropriate preparatory strategies, including the availability of advanced airway devices and involvement of experienced practitioners [18]. However, it is equally important to recognize that no single parameter can provide absolute certainty, and a multimodal assessment that combines several screening tools is likely to be the most effective strategy in routine practice.

When positioned within the broader literature, the results resonate with earlier meta-analyses that questioned the standalone value of conventional tests such as Mallampati grading and SMD. Nørskov et al. (2015) demonstrated that while these indices possess moderate predictive value, their diagnostic accuracy is compromised when applied independently [19]. Similarly, Faramarzi et al. (2018), in a large meta-analysis involving over 177,000 patients, reported the poor prognostic reliability of Mallampati assessment alone [20]. More recently, Chen et al. (2022) confirmed through systematic reviews and pooled data that advanced or ratio-based indices such as RHTMD and parameters derived from ultrasound imaging outperform single conventional predictors in both sensitivity and specificity [21]. These comparative observations suggest that airway assessment strategies are evolving towards incorporating multidimensional indices that adjust for anatomical variations across patient populations, which aligns with the findings of the present study.

From a clinical standpoint, the superiority of RHTMD in this study may be explained by its ability to normalize TMD relative to patient height, thereby reducing inter-individual variability that often limits the reproducibility of linear measures like SMD [22]. Mallampati grading continues to be widely applied due to its simplicity and ease of use, yet it is influenced by patient cooperation, posture, and examiner subjectivity, factors that have long reduced its predictive robustness. SMD, though anatomically relevant, is similarly influenced by head and neck mobility, limiting its consistency across different populations. The relatively weaker performance of AASI in this cohort may reflect differences in craniofacial morphology in Indian patients compared to Western populations, highlighting the need for population-specific validation of newer indices [23]. Point-of-care ultrasound has shown promising results in delineating soft tissue thickness, hyomental distances, and epiglottic visibility, parameters that may substantially improve the accuracy of difficult airway prediction [24,25]. In addition, the growing field of artificial intelligence offers opportunities to develop predictive models that can combine demographic, anthropometric, and imaging data to generate highly individualized risk scores [26]. The incorporation of such technological advances into routine preoperative evaluation could potentially transform airway assessment from a subjective exercise into a standardized, evidence-based process.

Despite these important insights, this study has certain limitations that must be acknowledged. The sample size was modest, with only 57 patients, which limits the statistical power and generalizability of the findings. Being a single-center study, the results may also be influenced by local demographic characteristics and operator experience. Furthermore, patients with higher body mass index and comorbid conditions were excluded, narrowing the scope of applicability. These limitations underscore the importance of larger, multicenter studies that encompass a more diverse patient population and incorporate a broader range of clinical settings. Future research should prioritize the integration of ultrasonography as a non-invasive and reproducible adjunct for airway assessment.

## Conclusions

This prospective observational study evaluated the predictive performance of several airway assessment parameters in identifying difficult endotracheal intubation among adults undergoing elective surgeries under general anesthesia. The findings highlight RHTMD as the most reliable single predictor, outperforming traditional measures such as MPC and SMD. However, the study also reinforces that relying on a combination of assessment tools provides superior diagnostic accuracy compared to any parameter used alone. Integrating multimodal airway evaluation into routine preoperative practice can greatly enhance preparedness, reduce the incidence of unanticipated difficult intubation, and ultimately improve patient safety. Continued research into emerging predictive modalities remains essential.

## Appendices

Appendix A 

**Table 4:** Study questionnaire ASA: American Society of Anesthesiologists The questionnaire was created by the authors.

Section	Parameter	Value
Patient information	Patient ID	
	Patient's age	
	Gender	
	ASA physical status [12]	
Baseline assessment	Modified Mallampati classification (MPC) [13]	
	Sternomental distance (SMD) [14]	
	Ratio of height to thyromental distance (RHTMD) [15]	
	Acromio-axillo-suprasternal notch index (AASI) [16]	
Outcome assessment	Cormack-Lehane grade	
Anesthetic management modifications	Alternative intubation technique	Yes / No
	Alternative airway device	Yes / No
	Surgical airway	Yes / No
	Other (specify)	
Airway complications	Hypoxia	Yes / No
	Aspiration	Yes / No
	Cardiac arrest	Yes / No
	Other (specify)	
Intubation Difficulty Score (IDS) [4]	Score (0-5)	
	Difficult intubation	Yes / No
